# Unmet health-related needs of heritable arrhythmogenic cardiomyopathy carriers in Belgium: The UR-HEART survey study

**DOI:** 10.1016/j.hroo.2025.08.029

**Published:** 2025-08-21

**Authors:** Phaedra Locquet, Eva Van Steijvoort, Pascal Borry, Zilke Claessens, Margaux Reckelbus, Tomas Robyns, Isabelle Huys

**Affiliations:** 1 Department of Pharmaceutical and Pharmacological Sciences, KU Leuven, Leuven, Belgium; 2 Department of Public Health and Primary Care, Centre for Biomedical Ethics and Law, KU Leuven, Leuven, Belgium; 3 Department of Cardiovascular Sciences, KU Leuven, Leuven, Belgium; 4 Department of Cardiovascular Diseases, University Hospitals Leuven, Leuven, Belgium

**Keywords:** Arrhythmogenic cardiomyopathy, Needs assessment, Unmet health-related needs, UR-HEART study, Patient survey

## Abstract

**Background:**

The revised European Pharmaceutical Legislation emphasizes research and development for unmet medical needs. While gene therapies for arrhythmogenic cardiomyopathies (ACM) are advancing, insight into patients’ lived experiences remains limited.

**Objective:**

This study identifies the unmet health-related needs of ACM carriers to guide policy, research, care, and treatment strategies.

**Methods:**

A cross-sectional survey was conducted among ACM carriers in Belgium using convenience sampling. The Needs Examination, Evaluation, and Dissemination (NEED) framework guided the development of a multilingual (Dutch, French, English) questionnaire assessing health-, health care- and social needs among symptomatic (S) and asymptomatic (A) carriers. Data were collected anonymously via Lime Survey or post. Descriptive and Wilcoxon signed-rank tests were conducted.

**Results:**

Of 112 participants (63 women, 49 men), most were over 40 years old (80%) and symptomatic (67%). Symptomatic carriers reported pain/discomfort (58%), daily activities limitations (51%), and anxiety/depression (49%), whereas asymptomatic carriers experienced anxiety/depression (30%). Despite high treatment satisfaction, half of participants found treatment burdensome, due to side effects (46%) and ongoing need to manage the condition (39%). Dissatisfaction centered on restrictions on competitive sports. Participants faced challenges with hobbies (S: 59%, A: 22%), reduced work intensity (S: 49%, A:19%), and financial consequences (S: 51%, A: 14%). Diagnostic delay of over 1 year persist (26%). Nearly half (45%) fulfilled their family planning before diagnosis, limiting informed reproductive choices. Moreover, 60% did not always receive useful information.

**Conclusion:**

Unmet needs among ACM carriers remain, particularly regarding delayed diagnosis, treatment burden, psychological stress, reproductive decisions, and access to information. Future care and research should address these gaps to improve ACM carriers’ quality of life.

## Graphical abstract

**Figure:**
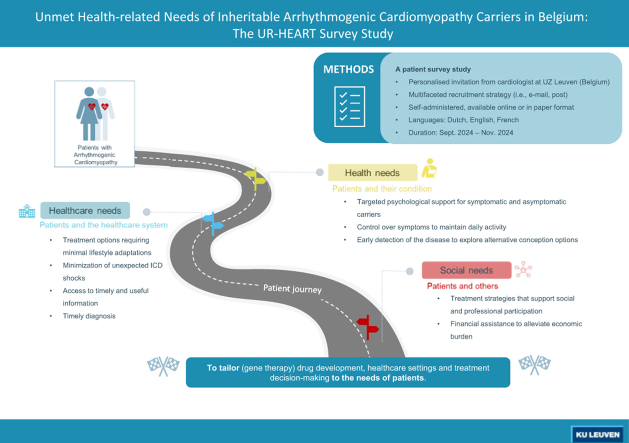

**Key Findings**

▪ Unmet health needs: The study reveals a decline in health-related quality of life after an arrhythmogenic cardiomyopathy diagnosis, with increased difficulties in daily activities, pain, and anxiety, even among asymptomatic carriers. Participants reported physical (fatigue, light-headedness) and psychological (fear, anxiety, and stress) symptoms, mainly experienced as mildly to moderately disturbing. With respect to family planning, many participants reported that they had already fulfilled their desire to have children by the time of diagnosis leaving them unable to make informed reproductive decisions.
▪ Unmet health care needs: While overall treatment satisfaction was high, invasive treatments were better tolerated than lifestyle modifications. Many carriers experienced life-threatening events before diagnosis, with delays of over 1 year being common, highlighting diagnostic challenges. Nearly all participants reported receiving appropriate care, but gaps in accessing timely and relevant information persist.
▪ Unmet social needs: Carriers reported challenges in daily activities, including difficulty in participating in hobbies, reduced work intensity, and financial consequences.

## Introduction

Arrhythmogenic cardiomyopathy (ACM) is a rare, autosomal dominant heart muscle disease that leads to progressive histological replacement of cardiomyocytes by fibrous or fibrofatty tissue, affecting the right, left, or both ventricles.^1^, ^2^, ^3^, ^4^, ^5^ Pathogenic variants occur in genes encoding both cardiac desmosomal, and non-desmosomal proteins.^6^ It is estimated that ACM affects around 1:2000 to 1:5000 people in the general population.^7^ However, due to under- and misdiagnosis, the actual prevalence is likely higher.^8^ Variable expressivity of ACM has been observed, with carriers presenting with symptoms at any age, typically between the third and fourth decade of life. The clinical manifestations range from palpitations and light-headedness to syncope, often triggered by ventricular arrhythmias.^3^^,^^5^^,^^9^^,^^10^ However, depending on the disease-causing genetic variant, sudden cardiac death (SCD) can be the first clinical manifestation, often occurring before the age of 30 years, during the concealed stage of disease.^8^

At present, (a)symptomatic carriers receive symptomatic management focused on preventing arrhythmias and SCD, alleviating heart failure symptoms and mitigating disease progression. This is achieved through various therapeutic interventions, including an implantable cardioverter defibrillator (ICD) implantation, pharmacological therapy, catheter ablation, lifestyle modification and, in severe cases, heart transplantation.^2^^,^^10^^,^^11^

Besides the physical impact, cardiogenetic conditions also have a significant impact on patients’ social lives and families.^12^ Individuals must often adjust their lifestyle, which can diminish quality of life (QoL), particularly in younger individuals who may struggle with mental well-being and experiences of anxiety and distress.^13^^,^^14^ This anxiety, combined with social isolation and reluctance to attend school or work, is further intensified by other lifestyle restrictions (eg, driver’s license restrictions, avoiding competitive sports).^15^^,^^16^ Depending on carriers’ genotype and phenotype, tailored recommendations are provided regarding participation in high-intensity endurance exercise and the continuation of athletic activities.^17^ These restrictions can furthermore lead to feelings of frustration, loss of autonomy, and anger.^15^^,^^16^

Due to its hereditary nature, ACM commonly affects multiple family members, with some experiencing SCD or other acute life-threatening cardiac events.^18^ These events bring high psychological distress, which is often intensified when pathogenic variants are also identified in other relatives. In such cases, carriers may feel responsible for passing on the disease, and may worry about other family members.^19^^,^^20^ Furthermore, as with other rare conditions, ACM carriers face various clinical challenges, including delayed diagnosis or misdiagnoses, and difficulties accessing appropriate care and treatment^21^^,^^22^

As illustrated, individuals affected by genetic cardiomyopathies face unmet health-related needs, spanning health, health care, and social dimensions, yet they are often overlooked by health care professionals.^23^^,^^24^

Understanding the unmet health-related needs of patients gained attention over recent years, especially in light of the revision of the European Pharmaceutical Legislation (2023), which aims to improve access to medicines that address unmet medical needs.^25^ In parallel, the Belgian ‘federal knowledge center for health care’ (KCE) has developed the ‘Needs examination, evaluation and dissemination’ (NEED) framework, a robust methodology to identify unmet health-related needs across medical conditions. The primary objective of this framework is to provide a tool to consider unmet needs of patients and society in policy decision-making regarding research and development, provision, and reimbursement of health interventions.^23^^,^^26^^,^^27^ This is particularly relevant for informing disease areas where patient-experience data is scarce, and where patients are limited in numbers.

To date, patient-centered evidence regarding the impact of ACM on carriers’ lives remains limited. Therefore, assessing their needs is a crucial first step to improve the care for ACM carriers.

## Methods

A cross-sectional patient survey was conducted using convenience sampling among individuals living with ACM in Belgium based on the NEED framework.^23^ It defines unmet health-related needs as condition-specific needs, categorized into 3 domains: (1) health needs, (2) health care needs, and (3) social needs. Each domain is evaluated using, (i) specific criteria to identify different type of needs, and (ii) indicators that assess the extent to which these needs are met or unmet. A need is perceived unmet when there is a gap between a patient’s observed health, reflecting current state with absent or existing, but possibly ineffective or suboptimal health interventions, and perfect health, which assumes no disease burden. Unmet needs are those not adequately fulfilled by current health, health care, and social interventions.

The survey conducted in this study represents the first step in the framework, aiming to guide further qualitative research. The follow-up phase will explore additional topics, such as gene therapy and health care delivery challenges, that were not addressed in the current study.

### Questionnaire development

The questionnaire was adapted from the generic version presented in the KCE report 348, with modifications to enhance its applicability to rare diseases, as described in the KCE report 377.^27^^,^^28^ This generic questionnaire was developed with items carefully selected based on a literature review on identifying unmet needs and drawn from psychometrically validated instruments.^23^^,^^28^ Furthermore, the questionnaire was adjusted and tailored to ACM based on input from a literature search and input from cardiologists, specifically experienced in the care of patients with ACM (n = 3), a representative from the Dutch patient organization PLN Foundation^29^ (n = 1), and members of a multi-stakeholder advisory committee composed of both ACM patient representatives and European Patients’ Academy fellows (n = 5).^30^ Additionally, the research team conducted an internal discussion to finalize the adjustments.

A pilot study involving 4 Dutch-speaking carriers was conducted to evaluate the clearness and applicability of the questionnaire. Based on the feedback received, several items within the questionnaire were refined, including expanding answer options to more accurately reflect the specific context of ACM.

The final survey consisted of 72 questions, covering: (1) Socio-demographic information, (2) Impact of ACM on work and education, (3) Experiences of symptoms (physical and/or psychological), (4) Time to diagnosis and treatment, (5) General health status before symptom onset and at present, (6) Impact of ACM on social life, (7) Other conditions, (8) Use and accessibility of health care, (9) Current and prior treatments, (10) Access to useful information, (11) Financial impact of ACM, and (12) Support network. The questionnaire furthermore included validated measures such as the European Quality of Life 5 dimensions 5 level (EQ-5D-5L) instrument to assess health-related QoL and Part II of the Nottingham health profile (NHP) to evaluate impact on social activities.^31^^,^^32^ Participants also rated the predictability of their condition on a scale from 1 (not very predictable) to 5 (very predictable). Similarly, participants rated their self-esteem on a scale from 1 (low self-esteem) to 5 (high self-esteem). The full survey is displayed in the Supplementary Materials.

The questionnaire was available in Dutch, English, and French, with each translation reviewed by native speakers.

### Participant recruitment

Eligible participants had to be diagnosed with a heritable form of ACM (carrier of a pathogenic or likely pathogenic variant according to the American College of Medical Genetics and Genomics/Association for Molecular Pathology criteria for variant interpretation in a definite ACM gene), over 18 years old, and residing in Belgium.^33^ A multifaceted recruitment strategy was employed to obtain a heterogenous sample reflecting variations in symptoms, demographic characteristics, and treatments received. Carriers deemed eligible for the study (n = 198) by a cardiologist (TR) from the University Hospital Leuven were contacted in September 2024. The questionnaire was distributed through e-mail and post when both contacts were available, allowing patients to choose their preferred method and facilitating participation by those with lower digital literacy.

Participants were asked to disseminate the survey with family members and acquaintances diagnosed with the same condition and complete it once either online through an online platform (Lime Survey Version 6.10.0) or on paper.^34^ A 3-week period was provided to respond, followed by a reminder extending the deadline by another 3 weeks.

Symptomatic carriers are defined as those who have self-reported physical symptoms, either currently or in the past, whereas asymptomatic carriers have not yet reported any such symptoms.

### Data collection

Conditional logic was applied in the online survey to ensure participants were only shown questions relevant to them based on their previous responses. Following the same logic, physical responses were manually entered in Lime Survey. For paper-completed questionnaires, in multiple-choice table format questions, if some options were checked while others were left blank, the blank fields were assumed to indicate that the respondent had not experienced the corresponding item. Free-text responses to ‘other’ questions were categorized into the original categories where applicable.

### Data analysis

Statistical analyses were performed using IBM SPSS Statistics 29. Descriptive analyses were used to describe characteristics for all items included in the questionnaire. For continuous variables, the mean, standard deviation (SD), and 95% confidence intervals (CIs) were calculated. For categorical variables, the corresponding valid percentages were calculated.

Self-reported health profiles were obtained prior to symptom onset and at present across the 5 EQ-5D-5L dimensions. From those individual health profiles, a health state index score was calculated using the latest Belgian value set, resulting in scores ranging from 1 (representing full health) to 0 (equivalent to a state as bad as death).^35^ The self-reported NHP scores were calculated by summing the number of positive responses, resulting in a score ranging from 0 (good) to 7 (bad).

Wilcoxon singed-rank tests were used to identify significant differences between scores measured at 2 time points (onset and present), as the assumption for normality was not met. A significance level of 0.05 was set for these statistical tests.

Results were presented for the total sample. Where applicable, separate percentages were calculated for symptomatic (S) and asymptomatic individuals (A). Full results are available in the Supplementary Materials.

## Results

### Socio-demographic characteristics

A total of 133 individuals accessed the questionnaire, of which 21 individuals were excluded from the analysis because they only answered socio-demographic questions, failed to meet the inclusion criteria, or provided similar answers from matching anonymized IP addresses. Table 1 presents 112 individuals (63 women, 49 men) that completed the questionnaire either online (58%) or on paper (47%). Most were over the age of 40 years (80%), had a higher level of education (60%), lived with a partner (70%), and were employed (54%). The most frequently observed pathogenic variants were in the Filamin C (26%), Desmoplakin (18%), and Lamin A/C (13%), while 26% were unaware of their causative gene. Additionally, 42% of participants had also other health issues, including conditions related to the locomotor system (34%), mental health (27%), and the digestive tract (25%).

**Table 1:** Socio-demographic characteristics of study participants

		n (valid %)
**Sex** (n = 112)	Women	63 (56.3)
	Men	49 (43.8)
**Largest age group (years)** (n = 112)	40-50	27 (24.1)
**Level of completed education** (n = 112)	No diploma/primary education	2 (1.8)
	Secondary education	32 (28.6)
	Higher vocational education	6 (5.4)
	Higher education of the short type (eg, bachelor’s degree)	30 (26.8)
	Higher education of the long type (eg, master’s degree)	30 (26.8)
	PhD	7 (6.3)
	Other (eg, adult education, certificates)	4 (3.6)
**Living situation** (n = 112)	Alone	20 (17.9)
	Alone with children	4 (3.6)
	Together with partner, without children	36 (32.1)
	Together with partner and children	42 (37.5)
	With parents or family	10 (8.9)
**Occupation at present** (n = 111)	Employee (physical work)	10 (9.0)
	Self-employed (physical work)	4 (3.6)
	Employee (office work)	39 (35.1)
	Self-employed (office work)	7 (6.3)
	Unemployed	1 (0.9)
	Retired	29 (26.1)
	Student	7 (6.3)
	Incapacitated	12 (10.8)
	Other (eg, a combination of office and physical work)	2 (1.8)
**Time since diagnosis** (n = 112)	More than 10 years	39 (34.8)
	5 to 10 years	25 (22.3)
	2 to 5 years	28 (25.0)
	6 months to 2 years	15 (13.4)
	2 months to 6 months	4 (3.6)
	I do not know	1 (0.9)
**Gene associated with their underlying pathogenic variant**∗ (n = 112)	PKP2	9 (8.0)
	DSG2	2 (1.8)
	DSP	20 (17.9)
	DES	4 (3.6)
	PLN	6 (5.4)
	FLNC	29 (25.9)
	LMNA	14 (12.5)
	Uncertain	29 (25.9)

∗ Multiple response question.

### Unmet health needs

Health needs were assessed across 3 criteria: (1) general health-related QoL, (2) physical and psychological health, and (3) reproductive health.

#### General health-related QoL

**Figure 1:**
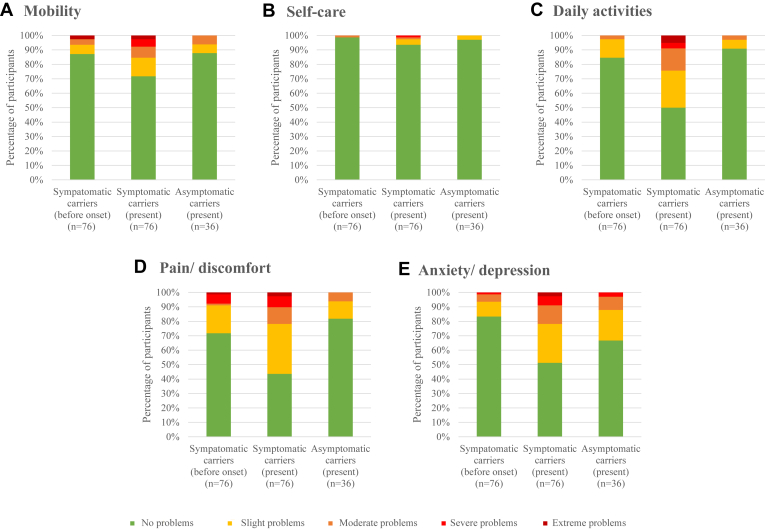
Self-reported level of problems on each EQ-5D-5L dimension of symptomatic carriers (before onset and present, n = 76) and asymptomatic carriers (present, n = 36).

Symptomatic carriers reported problems with pain/discomfort (58%), daily activities (51%) and anxiety/depression (49%), while asymptomatic carriers mainly experienced anxiety/depression (30%) and pain/discomfort (17%) (Figure 1).

Retrospectively, symptomatic carriers evaluated these dimensions comparing their condition before the onset of their first symptoms with their current stage. Across all dimensions, the proportion of health issues increased following the onset of symptoms. The most notable increases were seen in difficulties to perform daily activities (−35.7%), anxiety/depression (−31.5%), and pain/discomfort (−28.9%). The greatest increase in extreme problems was related to the ability to perform daily activities (−5.3%).

At the time of completing the survey, symptomatic carriers had a mean individual health stated index score of 0.75 (SD: 0.33, 95% CI: 0.67–0.83), while asymptomatic carriers scored 0.93 (SD: 0.11, 95% CI: 0.89–0.96).

Based on the retrospective ratings, a statistically significant decline in health index score was determined among 50 symptomatic carriers, with a decrease from 1 before the onset of their symptoms to 0.086 at the time of completing the questionnaire (z = −4.63, *P* < .001).

In addition, participants rated the predictability of their condition, resulting in a mean score of 2.18 ([SD: 1.26, 95% CI: 1.90 –2.46], S: 2.19, A: 2.00).

#### Physical and psychological health

**Figure 2:**
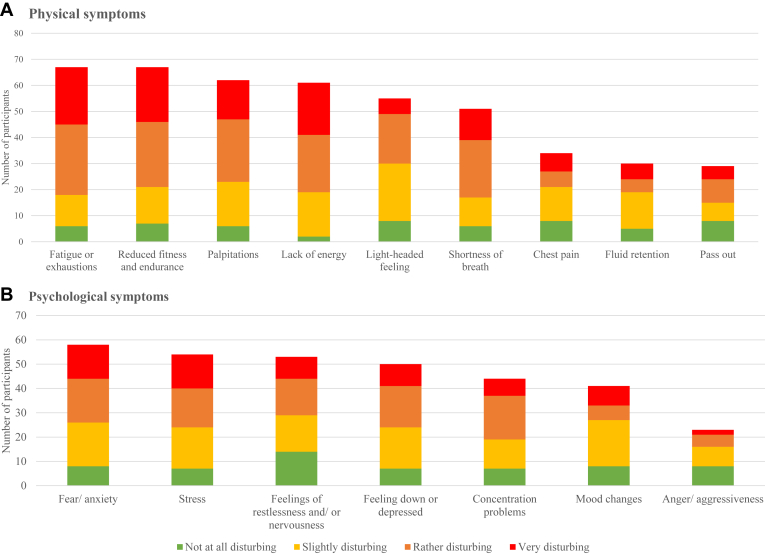
Self-reported frequency and level of disturbance of physical and psychological symptoms across the disease journey of symptomatic carriers (n = 75/76∗). ∗The results contain one missing value.

Among the participants (n = 112), 67% reported physical symptoms and 59% reported psychological symptoms. Among those who experienced physical symptoms, the most commonly reported symptoms were fatigue or exhaustion (88.2%), reduced fitness and endurance (88%), palpitations (80%), lack of energy (80%), light-headed feeling (72%) and shortness of breath and breathing difficulties (67%) (Figure 2). Symptoms most frequently considered to be very disturbing, included reduced fitness and endurance (31%), fatigue or exhaustions (33%) and lack of energy (33%).

Fear/anxiety (88 %), stress (82%), feelings of restlessness/nervousness (80%), and feelings of being down/depressed (76%) were most often reported. Of these, fear/anxiety (24.1%) and stress (25.9%) were most frequently considered to be very disturbing (Figure 2).

Symptomatic and asymptomatic carriers rated their self-esteem with mean scores of 3.40 (SD: 0.89; 95% CI: 3.20–3.61) and 3.56 (SD: 1.04; 95% CI: 3.19–3.94). A statistically significant decline in self-esteem was revealed among symptomatic participants, with a decrease of −0.5 following the onset of their symptoms (z = −4.067, *P* < .001).

#### Reproductive health

**Figure 3:**
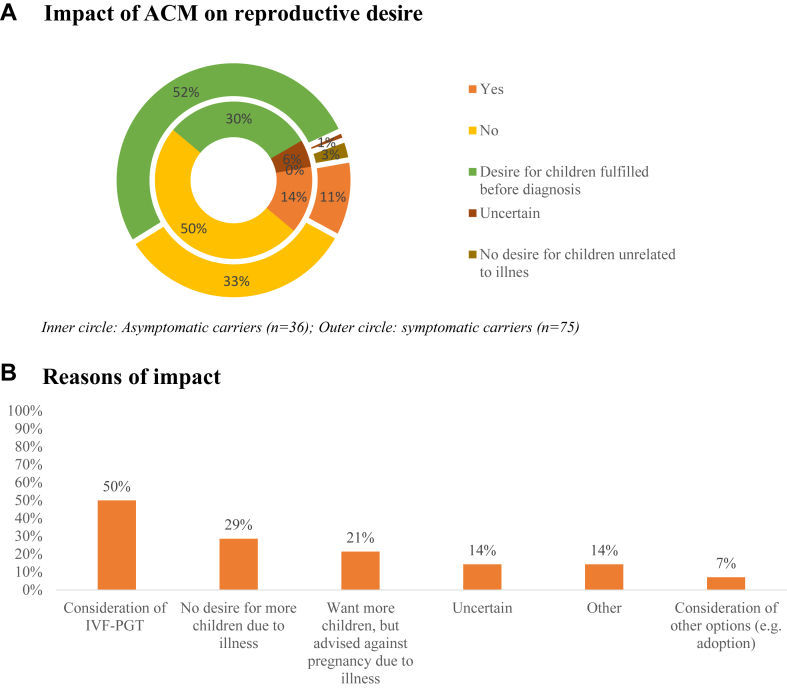
Percentage of participants reporting an impact of ACM on their reproductive desire (n = 111) and the underlying reasons∗ (n = 14). Results related to survey questions: a. “Has your illness influenced your desire to have children?,” b. “If so, in which way?”. ACM = arrhythmogenic cardiomyopathy; IVF-PGT = in vitro fertilization preimplantation genetic testing. ∗Multiple answer question.

Before receiving their ACM diagnosis, 45% of participants (n = 111) had already fulfilled their desire to have children. For 12% (n = 13, S: 10.7%, A: 13.9%), their condition had an impact on their desire to have children, leading them to opt for in vitro fertilization combined with preimplantation genetic testing (50%) (Figure 3).

### Unmet health care needs

Health care needs were assessed across 4 specific criteria: (1) treatment use, (2) satisfaction and burden of treatment, (3) quality of care, and (4) accessibility of care.

#### Treatment use

Most participants had already received some form of treatment (n = 94, 82%). The most common strategies, reported by over 50% of them, included the administration of prescription drugs (87%), lifestyle adjustments (71%), use of medical devices (ie, ICD, pacemaker) (70%), and undergoing surgical interventions (53%). More specifically, among those receiving treatment (n = 92), the majority had previously undergone an ICD implantation (64%), catheter ablation (39%), or a heart transplant (23%). Within the last 2 years, most participants had been treated with beta-blockers (72%), statins (37%), and mineralocorticoid receptor antagonist (24%).

Overall, participants (n = 93) self-reported a high level of treatment adherence, with 85% stating that they always take their medication as prescribed by their doctor. Furthermore, most participants (n = 93) assessed their treatments as effective (50%), while 26% reported that it was only effective to a certain extent. Additionally, 56% of participants were satisfied with their current level of involvement in treatment decisions for ACM and did not wish to be more involved.

#### Satisfaction and burden of treatment

**Figure 4:**
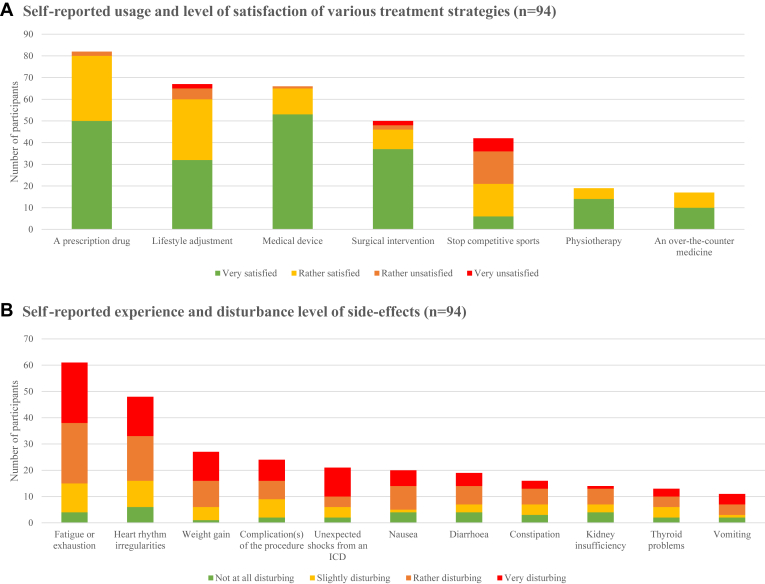
Percentage of participants reporting usage, satisfaction and disturbance related to various treatment strategies and their associated side-effects (n = 94). Results related to survey question(s); a. “What treatments are you receiving (or have you received) for your genetic heart muscle disease? And how satisfied are you with these?,” b. “If you experience(d) any side effects when treating your genetic heart muscle disease, indicate to what extent each of these are/were disturbing.” ICD = implantable cardioverter defibrillator.

Overall, participants who had previously received treatment expressed the highest levels of satisfaction with management strategies such as medical devices and prescription drugs. In contrast, participants reported the greatest dissatisfaction with strategies involving the cessation of competitive sports and lifestyle adjustments (Figure 4). When asked about the overall burden of their treatment (n = 94), most participants characterized the treatment(s) they received as either not burdensome (42%) or slightly to extremely burdensome (50%). Among the 50% who found their treatment burdensome, along with the 7% who were unsure, the main reasons included side effects of the treatment (46%), the continuous need to manage their illness and treatment (39%), and the strict lifestyle discipline it requires (35%).

Furthermore, 81% of participants reported experiencing side effects, with fatigue or exhaustion (65%) and heart rhythm irregularities (51%) occurring most frequently. Of these, weight gain (78%), nausea (75%), fatigue or exhaustion (74%) and unexpected shocks from an ICD (71%) were experienced as most disturbing (Figure 4).

#### Quality of care

**Figure 5:**
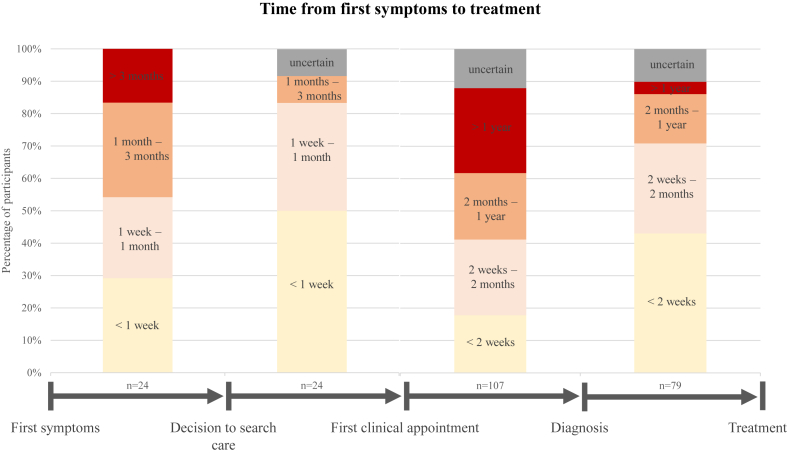
Time from first symptoms to diagnosis and treatment. Results related to survey question(s): 1. “How much time passed between your first symptoms and your decision to see a doctor? (By ‘first symptoms’ we mean symptoms you may not have realized were abnormal at that time.),” 2. “How much time passed between your decision to see a doctor and the first consultation?, 3. “How much time passed between the first visit to the doctor and the diagnosis?,” 4. “How much time passed between diagnosis and receiving your first treatment (surgical or drug)?”

Most participants (57%) had been diagnosed with ACM for more than 5 years. When asked how they discovered they had ACM, 46% were diagnosed following genetic testing prompted by a family member’s diagnosis, 23% consulted their doctor after experiencing the first symptoms, and 17% were diagnosed upon emergency room admission, where an abnormality was discovered. Among those who consulted their doctor, most patients took up to 1 month (54%) to decide to seek medical advice, after which most were able to see a doctor within a week (50%). Overall, 47% of participants waited more than 2 months to receive a diagnosis after their first visit, with 26% receiving a diagnosis only after more than 1 year, regardless of how the condition was discovered. Among those currently receiving treatment, the majority began treatment within less than 2 months after they had received their diagnosis (71%) (Figure 5).

Most participants (n = 112) reported having sought care from a specialist doctor (91%; S: 93%, A: 86%), a general practitioner (75%; S: 82%, A: 61%), and the emergency department (33%; S: 41%, A: 17%) due to their condition. Participants (n = 111) were asked about the extent to which they had received useful information from their health care providers, with only 41% (S: 45%, A: 31%) reporting that they always received timely and useful information. Doctors and specialists (90%; S: 92%, A: 86%) and medical websites/apps (18%; S:21%, A: 11%) were reported as the most useful sources of information in general.

#### Accessibility of care

Almost all participants (96%; S: 96%, A: 94%) indicated that they had received appropriate care when needed in the past 12 months. Additionally, when asked if they needed extra support that was not provided, the majority of participants indicated that they did not experience any unmet support need (51%; S: 43%, A: 69%). However, others indicated a need to consult other health professionals, such as a psychologist (19%; S: 23%, A: 11%), and 16% (S: 20%, A: 8%) desiring to connect with other patients who have the same condition, particularly among symptomatic individuals.

Moreover, participants were asked if they had difficulty finding someone they could trust to discuss their condition with. Most participants either did not experience difficulties in finding someone (50%; S: 51%, A: 46%) or felt they did not need such a person (29%; S: 25%, A: 37%).

### Unmet social needs

Social needs were assessed across 4 specific criteria: (1) social life, (2) education, (3) work and financial situation, and (4) stigma.

#### Impact on social life

**Figure 6:**
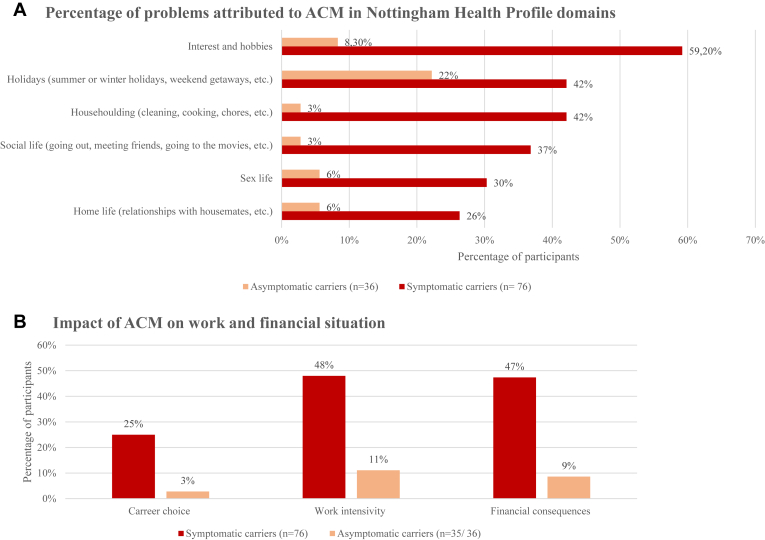
Impact of ACM on social life, work, and financial situation. Results related to survey question(s): a. “Has your health condition caused problems with your:,” b. “Has your genetic heart muscle disease had a financial impact? If yes, what kind of impact?.” ACM = arrhythmogenic cardiomyopathy.

On average, participants had a NHP score of 1.76 (SD: 2.01, 95% CI: 1.38–2.14), with the most problems experienced by symptomatic carriers in areas such as interest and hobbies (47%; S: 59%, A: 22%), holidays (31%; S: 42%, A: 8%) and householding (30%, S: 42%, A: 6%) (Figure 6).

#### Impact on education

The majority of participants (88%; S: 87%, A: 88%) indicated that they had not missed any educational activities because of their condition.

#### Impact on work and financial situation

Participants (n = 112) were asked if their condition had impacted their career choice, with 78% (S: 71%, A: 92%) reporting that it had no impact. Among the remaining 22%, the most frequently cited reasons were categorized as “other” (36%) including; discontinuing self-employment and needing to stop working because of their condition. Additionally, participants were asked if ACM had influenced their work intensity. No impact was reported by 60% (S: 51%, A: 81%), while the remaining 40% most commonly cited a reduction in working hours (42%) as the primary adjustment. Among participants who were working at the time of completing the survey, the average number of days absent from work in the past 12 months was 2.4 days (SD: 6.64, 95% CI: 0.64–4.16). For those who reported taking early retirement (12%; S: 14%, A: 0%), the average retirement age was 52 years (SD: 8.18, 95% CI: 31.67–72.33). Eleven participants (10%; S: 13%, A: 3%) reported being unable to work, that is, incapacitated at the time of completing the survey, an occupational status they did not had prior to their diagnosis, as they were employed before.

Participants were asked if they had experienced a financial impact due their condition. No impact was reported by 60% (S: 49%, A: 86%). Among those who did report an impact, the most common reasons were loss or lack of income (55%) and medical expenses (55%).

#### Stigma

Participants reported almost never experiencing stigmatization (51%, S: 44%, A: 67%) due to their condition, while others reported they experienced it rarely (20%, S: 24%, A: 11%) or occasionally (17%, S: 15%, A: 9%).

## Discussion

This study represents the first needs assessment of ACM carriers identifying the dimension of life that are most affected by the condition.

Our results indicate a decline in health-related QoL (HRQoL) following an ACM diagnosis, even among asymptomatic carriers. Participants reported physical (fatigue, light-headedness) and psychological symptoms (fear, anxiety, and stress), mainly experienced as mildly to moderately disturbing. Challenges in daily activities were observed, particularly among symptomatic individuals. These included difficulties engaging in hobbies, reduced work intensity, and financial consequences.

Overall, treatment satisfaction was high, with invasive treatments better tolerated than those requiring lifestyle modifications. Diagnostic delays of over 1 year persist, with some requiring emergency room admission before diagnosis. With respect to family planning, many participants reported that they had already fulfilled their desire to have children by the time of diagnosis. Finally, while almost all participants reported receiving appropriate care when needed, they also reported not always receiving timely and useful information.

### Unmet need for psychological support and treatment optimization

The reduced HRQoL observed in this study is consistent with previous research showing lower mental and physical QoL among both ACM carriers in the United States and patients with ICD with other cardiac conditions.^16^^,^^36^^,^^37^ Suárez Bagnasco et al^38^ assessed the psychological impact of heritable cardiomyopathies indicating that younger age, functional capacity, and ICD shocks contribute to both device-specific and general health-related anxiety. Our findings confirm that ACM carriers experience anxiety and find unexpected ICD shocks to be disturbing, suggesting this as a likely contributing factor to their observed decline in HRQoL. Moreover, we also observed a reduced HRQoL in asymptomatic carriers, possibly due to fears related to hereditary diseases, as noted by Friess et al.^39^ Considering the psychosocial impact of ACM, the significant number of participants seeking additional psychological support, and the established link between anxiety, ventricular arrhythmias, and mortality in patients with ICD, there seems to be an unmet need to: (1) identify those at risk for anxiety, (2) provide targeted psychological support for symptomatic and asymptomatic carriers, (3) minimize unexpected ICD shocks, and (4) explore alternative treatment options.^38^^,^^40^

Remarkably, our study results show higher satisfaction with medical devices compared to treatments that require lifestyle changes. In general, many found their treatment burdensome, primary due to side effects and the ongoing need to manage both their illness and treatment. Qualitative insights from Schopp et al^41^ further support these results, with participants expressing dissatisfaction over changes in their social lives due to the inability to participate in sports. Consequently, there is a need for alternative treatments that allow carriers to maintain their lifestyle with minimal disruption.

### Unmet need for timely diagnosis and accessible information

Many carriers in this study faced diagnostic delays, which is consistent with an Italian study that observed a significant diagnostic delay of ≥2 years to receive an ACM diagnosis.^42^ Diagnosing ACM, especially in its early stages, is widely recognized as challenging, leading to frequent updates in diagnostic criteria.^5^^,^^43^

In contrast to neighboring countries, Belgium does not have formally recognized rare disease centers, leaving patients to navigate between hospitals, which often results in delays in diagnosis and treatment.^44^, ^45^, ^46^ The establishment of specialized centers for rare conditions in Belgium could help address this gap, along with the potential expansion of genetic testing for patients with cardiac-related issues.

Some studies suggest that predictive genetic testing for at risk individuals does not impact their HRQoL, while others indicate that hereditary disease-related fears can reduce their HRQoL.^39^^,^^47^ Additionally, genetic testing in minors for ACM is complex due to its variable phenotypic expression, and the current absence of preventive interventions. While early identification could offer benefits, such as closer monitoring, it also raises concerns about psychological harm, social discrimination, and autonomy.^48^^,^^49^ The debate is further complicated by the high risks of SCD before the age of 30 years in ACM carriers, highlighting the urgent need to prevent life-threatening events.

Most of our participants had already fulfilled their desire to have children by the time of their diagnosis, limiting informed reproductive choices. This temporal conflict can include the discovery and testing of their children for ACM, as well as the risks of SCD in potentially affected but undiagnosed children, which could add psychological distress. These reproductive considerations and the risk of SCD underscore the need for timely diagnosis.^2^ This could improve disease management, reduce life-threatening events, and enable carriers to make more informed reproductive decisions.

Our participants trusted doctors and specialists as the most reliable sources of information, but more than half of our participants still experienced an informational gap. There is currently no ACM-specific patient organization in Belgium, which could provide valuable additional support and information alongside health care providers. Given the identified unmet need for accessible information in this study, we see an opportunity to improve: (1) access to reliable resources, (2) patient education, and (3) the establishment of dedicated patient organizations.

### Unmet need for social and financial support

Our study results furthermore indicate that ACM impacts not only physical health but also limits participation in activities that enhance QoL and personal fulfillment. Our findings align with those of Schopp et al,^41^ who also identified significant changes in social activities that affect the daily lives of carriers. Carriers adapted their lifestyles from the moment of diagnosis, underscoring the extensive impact of the condition beyond medical considerations.^41^

Moreover, Etchegary et al^50^ examined the economic burden on families affected by ACM, reinforcing our study findings that employment and career choices are regularly influenced by the condition, alongside concerns related to insurance. Furthermore, Allan et al^51^ highlighted that financial burdens extend to families who experience SCD of a relative, often due to loss of income or the need to take unpaid leave to manage grief and funeral planning. These financial stressors may also be relevant to ACM carriers, considering the high risk on SCD associated with the condition.^51^ Considering the substantial social and financial impact observed in our study, we emphasize the need for: (1) treatment strategies that support social and professional participation, and (2) financial assistance to alleviate the associated economic burden.

The unmet needs identified in this study may be partly attributed to the reported unpredictability of ACM, as well as the physical and psychological symptoms that impact daily life due to both the condition itself and its treatments. Given the progressive nature of ACM, these challenges are likely to intensify over time, further affecting patients’ QoL.^52^ Currently, no treatment strategy fully addresses these challenges. However, advancements in the development of gene therapies offer potential benefits, including the possibility of halting disease progression, or in some cases, providing preventive treatment.^53^ A recent study of Schopp et al^41^ (2024), reported that ACM carriers showed enthusiasm for gene therapy trials, with interest varying based on the experienced disease severity, personal circumstances, and trial-specific factors such as risk, benefits, and study burden. Nonetheless, it remains unclear whether these therapies would fully address the unmet needs identified in this study, though the findings highlight which needs should be carefully considered when implementing future treatments.

## Limitations

The use of a survey might have limited the identification of unanticipated needs. Although an open-ended question was included, a follow-up qualitative study is suggested to explore this further. The questionnaire, while developed with experts and patient input, was not formally validated, and its reliability remain unconfirmed.

The small sample size and single-center design limit the generalizability of our findings. Furthermore, it may have led to the inclusion of participants from the same family potentially introducing a certain bias. Participants were asked to share the survey with others diagnosed with the same condition, which could have resulted in the inclusion of individuals who did not fully meet the inclusion criteria. To mitigate this, we incorporated screening questions to exclude ineligible participants. The study’s reliance on self-reported symptoms and genetic diagnosis limited clinical depth. The older than usual cohort and low prevalence of PLN variants, despite the Belgian context, may have influenced insights into lifestyle and reproductive decisions. Our study included heart transplant recipients, who may experience improved health post-transplant. As a result, their responses may not fully reflect the experiences of those in more advanced stages, potentially leading to an underrepresentation of this group. Furthermore, the reliance on self-reported data introduces the possibility of recall and social desirability bias, particularly as 35% were diagnosed over 10 years ago, specifically affecting responses on pre-symptom QoL and timing of care.

Both symptomatic and asymptomatic carriers were included, though we acknowledge that, as with individuals with varying disease severity, they may have different needs. Due to the limited sample size, statistical comparisons were not conducted. Future research should directly compare these groups; including a healthy control group, to provide a better understanding of their unique experiences and challenges. Psychological symptoms in asymptomatic carriers were not assessed, though QoL data suggest they may exist. Finally, data entry from paper surveys into the dataset revealed inconsistencies between shifting questions, and follow-ups. For example, in the shifting question: “Are you being or have you been treated, […]?,” where a participant answered “No.,” but the follow-up —one that would not have been visible to an online participant—suggested they had received treatment. This suggests a lack of clarity in the questions and potential data loss. Unanswered items in the multiple-choice table format were interpreted as the respondent having not experienced the corresponding items, which may have led to misclassification bias. Additionally, some questions on paper remained unanswered, leading to missing data.

## Conclusion

This study presents the significant impact of ACM on carriers’ daily lives, affecting their physical, psychological, and social well-being. Despite symptomatic treatments, significant unmet needs remain, particularly concerning delayed diagnosis, reproductive decision-making, treatments strategies that disrupt daily life, psychological burden, and limited access to timely information. The condition’s unpredictable and progressive nature may further exacerbate these challenges. Our study findings can guide future research initiatives and contribute to the responsible development and implementation of treatment and health care strategies for ACM carriers.

Future efforts should focus on improving early diagnosis through genetic screening and the establishment of specialized centers, strengthening support networks and psychological care, and exploring emerging treatments, such as gene therapy, to address the long-term impact of ACM on carriers’ health and QoL.

## Disclosures

During the preparation of this work the author(s) used GTP-4o mini in order to improve the readability and language. After using this tool/service, the author(s) reviewed and edited the content as needed and take(s) full responsibility for the content of the publication.

## Footnotes

Appendix Supplementary data associated with this article can be found in the online version at https://doi.org/10.1016/j.hroo.2025.08.029.
