# Supplementary material for: Unmet health-related needs of heritable arrhythmogenic cardiomyopathy carriers in Belgium: The UR-HEART survey study
Source: Heart Rhythm O2. 2025 Aug 21;6(11):1773–85. doi: 10.1016/j.hroo.2025.08.029 (PMC12675060; doi:10.1016/j.hroo.2025.08.029)
Supplement: Supplementary material [file mmc1.pdf]

Supporting information

Unmet Health-related Needs of Inheritable Arrhythmogenic Cardiomyopathy Carriers in Belgium:

The UR-HEART Survey Study

Locquet P, Van Steijvoort E, Borry P, Claessens Z, Reckelbus M, Robyns T, Huys I**

Table of Contents

Supplementary material I: Patient survey 2

Supplementary material II: Additional tables 3

Table 1: Sociodemographic characteristics of participants 3

Table 2: Self-reported level of problems on each EQ-5D-5L dimensions of symptomatic carriers (S) (before onset and present, n=78) and asymptomatic carriers (A) (present, n=33). 5

Table 3: Change in self-reported level of problems (% before onset - % present) on each EQ-5D-5L dimension of asymptomatic carriers (n=78). 5

Table 4: Mean EQ-5D-5L health index scores of symptomatic carriers (before onset and present, n=78) and asymptomatic carriers (present, n=33). 6

Table 5: Self-reported frequency and level of disturbance of physical symptoms (n= 77/78). 6

Table 6: Self-reported frequency and level of disturbance of psychological symptoms (n=77/78). 6

Table 7: Impact of ACM on reproductive decision-making of participants and underlying reasons. 7

Table 8: Self-reported frequency and level of satisfaction of treatment strategies. 7

Table 9: Frequency of surgical interventions over disease course (n=92) and drugs usage of past two years (n=82) 7

Table 10: Self-reported treatment effectiveness, burden and adherence. 8

Table 11: Self-reported frequency and level of burden of side-effects (n=77/78). 9

Table 12: Type of healthcare provides participants have been in contact with for their ACM. 9

Table 13: Receival of useful information at the moment carriers need it and the source of most useful information. 10

Table 14: Pathway and timing to diagnosis and treatment. 10

Table 15: Reported need for additional support that was not received (n=112). 11

Table 16: Areas of NHP domains were participants experienced problems with (n=112). 11

Table 17: Impact on career choice and work intensity. 12

Table 18: Reported financial impact and reasons of impact. 13

Table 19: Reported experience of stigmatization 13

Table 20: Years of education lost due to ACM 13

Supplementary material I: Patient survey

A QUESTIONNAIRE

What are your needs as a patient with a genetic heart muscle disease?

Thank you for participating in our survey.
Our study aims to identify the needs of patients with genetic myocardial diseases to improve their care.

YOUR NEEDS AND WISHES ARE OUR PRIORITY!

UZ LEUVEN

KU LEUVEN

We kindly ask that you complete the questionnaire only once. If you already have the questionnaire via email received and completed online, you do not need to send it again by post.

Please remember that your participation is entirely anonymous.

Additionally, we would greatly appreciate it if you could share this survey with yourfamily and fr iends. This will help us reach as many people as possible who have a genetic heart muscle disease.

You can read more information and background about the study in the attached information lett er. This study was approved by the UZ/KU Leuven research ethics committee.

If you prefer to complete the questionnaire online, you can do so via the following QR code:

Contact [X] for your questions regarding this study.
Tel. [X], email: [X]

Section A: General information

In this section we would like to find out more about your background and experiences regarding education, profession and howc your illness may have influenced this.

A1.b Since how long approximately have you been diagnosed with your genetic heart muscle disease?

- Less than 2 months ☐
- 2 months to 6 months ☐
- 6 months to 2 years ☐
- 2 to 5 years ☐
- 5 to 10 years ☐
- More than 10 years ☐
- I have not been diagnosed by a doctor ☐
- I do not know ☐

A2. Which gene is responsible for your genetic heart muscle disease? (Multiple answers possible)

- PKP2 (Plakophilin C) ☐
- DSG2 (Desmoglein 2) ☐
- DSP (Desmoplakin) ☐
- DSC2 (Desmocollin 2) ☐
- DES (Desmin) ☐
- PLN (Phospholamban) ☐
- CDH2 (cadherin-2) ☐
- FLNC (Filamin C) ☐
- LMNA (Lamin A/C) ☐
- TMEM43 (Transmembrane protein 43) ☐
- I do not know ☐
- Other ☐

Other

A3. What is your biological gender?

- Male ☐
- Female ☐
- Other ☐

Other

A4. Please choose your age bracket/ group.

- [ ] Younger than 18 years
- [ ] 18-25 years
- [ ] 25-30 years
- [ ] 30-40 years
- [ ] 40-50 years
- [ ] 50-60 years
- [ ] 60-70 years
- [ ] Older than 70 years

A5. What country do you live in?

- [ ] Belgium
- [ ] The Netherlands
- [ ] Other

Other

A6. You live:

- [ ] Alone
- [ ] Alone with children
- [ ] Together with partner, without children
- [ ] Together with partner and children
- [ ] With parents or family
- [ ] In a residential and care center
- [ ] Other

Other

A7. What is the highest diploma or degree you have obtained so far?

- No diploma/primary education ☐
- Secondary education ☐
- Higher vocational education (HBO5) ☐
- Higher education of the short type(e.g. bachelor's degree) ☐
- Higher education of the long type (e.g. master's) ☐
- PhD ☐
- I do not know ☐
- Other ☐

Other

Section B:

B1. **What was your main professional status <u>just before</u> the diagnosis of your genetic heart muscle disease?**

- Employee (mainly physical work) ☐
- Employee (mainly office work) ☐
- Self-employed (mainly physical work) ☐
- Self-employed (mainly office work) ☐
- Unemployed ☐
- Student ☐
- Retired ☐
- Incapacitated ☐
- Other ☐

Other

B2. **What is your main professional status <u>at this moment</u>?**

- Employee (mainly physical work) ☐
- Employee (mainly office work) ☐
- Self-employed (mainly physical work) ☐
- Self-employed (mainly office work) ☐
- Unemployed ☐
- Student ☐
- Retired ☐
- Incapacitated ☐
- Other ☐

Other

B3. Has your genetic heart muscle disease influenced your career choice?

- Yes ☐
- No ☐
- Don't know ☐

B4. If so, in what way? (Several answers possible)

- I have accepted a lower-paying position ☐
- I have had to reject promotions ☐
- I ended up in a different sector than I originally planned ☐
- I chose a profession with flexible hours to better manage my health ☐
- I chose a profession that is less physically demanding ☐
- I do not know ☐
- Other ☐

Other

[ ]

B5. Has your genetic heart muscle disease affected the intensity/ productivity(number of working hours, responsibilities) of your work? (Several answers possible)

- Yes ☐
- No ☐
- I do not know ☐

B6. If so, in what way? (Several answers possible)

- Reducing of working hours ☐
- Frequently taking unpaid leave ☐
- Frequenly taking sick leave ☐
- Loss of employment ☐
- Taking early retirement ☐
- I do not know ☐

Other ☐

Other

B7. **How many working days in total have you been absent from work due to your genetic heart disease in the past 12 months? If you are unable to indicate this number of days correctly, please provide an estimate.**

B8. **If you took early retirement: At what age was this?**

B9. **How many months or years of education have you lost as a result of your genetic heart muscle disease?**

- Less than 6 months ☐
- 6 months to 1 year ☐
- 1 to 2 years ☐
- More than 2 years ☐
- I have not missed any education ☐
- I do not know ☐

Section C: About your condition

In this section we would like to learn more about your symptoms, the time it took for the diagnosis, any hospitalizations and other aspects related to your health situation.

C1. **Have you, either now or in the past, experienced any physical and/ or psychological symptoms related to your chronic heart muscle disease?**

- Yes ☐
- No ☐

C2. Which of the following symptoms are you currently experiencing, or have you previously experienced, related to your genetic heart muscle disease? How disturbing were they? (You do not have to indicate side effects of your treatment here)

	Very disturbing	Rather disturbing	Slightly disturbing	Not at all disturbing	I have never experienced this symptom before
Reduced fitness and endurance	☐	☐	☐	☐	☐
Shortness of breath and breathing difficulties	☐	☐	☐	☐	☐
Fatigue or exhaustion	☐	☐	☐	☐	☐
Lack of energy	☐	☐	☐	☐	☐
Light-headed feeling	☐	☐	☐	☐	☐
Pass out	☐	☐	☐	☐	☐
Palpitations	☐	☐	☐	☐	☐
Chest pain	☐	☐	☐	☐	☐
Edema/swelling/fluid retention	☐	☐	☐	☐	☐
Concentration problems	☐	☐	☐	☐	☐
Mood changes	☐	☐	☐	☐	☐
Fear/ Anxiety	☐	☐	☐	☐	☐
Feeling down or depressed	☐	☐	☐	☐	☐
Stress	☐	☐	☐	☐	☐
Anger / Aggressiveness	☐	☐	☐	☐	☐
Feelings of restlessness and/or nervousness	☐	☐	☐	☐	☐

C3. Do you experience any other symptoms? If so, what are they and how bothersome are they for you?

C4. If you have been diagnosed, but you do not have any symptoms at the moment, do you know if there is a present affect upon your heart because of your disease, e.g. visibile on your ECG, echo or MRI?

- Yes, my heart is already affected ☐
- Yes, but I don't know exactly how or to what extent ☐
- No, my heart is not yet affected ☐
- I do not know ☐

C5. **If so, can you clarify how and to what extent it is already affected?**

Section D:

D1. **How did you discover that you have a genetic heart muscle disease? Select the option that best fits your situation:**

- I visited my doctor after experiencing symptoms related to the disease. ☐
- An abnormality in my heart was accidentally discovered during a medical check-up or a regular doctor's appointment. ☐
- An abnormality in my heart was discovered after an emergency admission to a hospital. ☐
- Through genetic screening after a family member was diagnosed with the disease. ☐
- I do not know ☐
- Other ☐

Other

D2. **How much time passed between your first symptoms and your decision to see a doctor? (By 'first symptoms' we mean symptoms you may not have realized were abnormal at that time.)**

- Less than 1 week ☐
- 1 week to 1 month ☐
- 1 month to 3 months ☐
- More than 3 months ☐
- I had no symptoms before my first consultation ☐
- I do not know ☐

D3. **How much time passed between your decision to see a doctor and the first consultation?**

- Less than 1 week ☐
- 1 week to 1 month ☐
- 1 month to 3 months ☐
- More than 3 months ☐
- I do not know ☐

D4. **How much time passed between the first visit to the doctor and the diagnosis?**

- Less than 2 weeks ☐
- 2 weeks to 2 month ☐
- 2 months to 1 year ☐
- More than 1 year ☐
- I do not know ☐

D5. **How much time passed between diagnosis and receiving your first treatment (surgical or drug)?**

- Less than 2 weeks ☐
- 2 weeks to 2 months ☐
- 2 months to 1 year ☐
- More than 1 year ☐
- I'm not getting treatment ☐
- I do not know ☐

D6. **On a scale of 1 to 5, how would you rate the predictability of your disease? Take into account the possibility of sudden changes, unclear signs of improvement or decline, and the difficulty of planning ahead? (1 is not very predictable, 5 is very predictable)**

- 1 ☐
- 2 ☐
- 3 ☐
- 4 ☐
- 5 ☐

D7. **How many times have you been hospitalized for 1 night or more because of your genetic heart muscle disease?**

Section E: Your general health

We would like to know your current state of health and how you experienced it before the symptoms of your genetic heart muscle disease appeared (if applicable). Please check the box next to the statement that best describes your health before the first symptoms of your genetic heart muscle disease appeared (if applicable) and how you experience your general health today. (**If you are not currently experiencing any symptoms, you only need to complete the questions that assess your health today.**)

E1. Your mobility before the symptoms of your genetic heart muscle disease occurred

- I had no problems in walking about ☐
- I had slight problems in walking about ☐
- I had moderate problems in walking about ☐
- I had severe problems in walking about ☐
- I was unable to walk about ☐

E2. Your mobility today

- I have no problems in walking about ☐
- I have slight problems in walking about ☐
- I have moderate problems in walking about ☐
- I have severe problems in walking about ☐
- I am unable to walk about ☐

E3. Your self-care before the symptoms of your genetic heart muscle disease occurred

- I had no problems washing or dressing myself. ☐
- I had slight problems washing or dressing myself. ☐
- I had moderate problems washing or dressing myself. ☐
- I had severe problems washing or dressing myself. ☐
- I was unable to wash or dress myself. ☐

E4. Your self-care today

- I have no problems washing or dressing myself. ☐
- I have slight problems washing or dressing myself. ☐
- I have moderate problems washing or dressing myself. ☐
- I have severe problems washing or dressing myself. ☐
- I am unable to wash or dress myself. ☐

© EuroQol Research Foundation. EQ-5D™ is a trade mark of the EuroQol Research Foundation

E5. Your daily activities <u>before</u> the symptoms of your genetic heart muscle disease occurred (e.g. work, study, sports, housekeeping, family and leisure activities)

- I had no problems doing my daily activities. ☐
- I had slight problems doing my daily activities. ☐
- I had moderate problems doing my daily activities. ☐
- I had severe problems doing my daily activities. ☐
- I was unable to do my daily activities. ☐

E6. Your daily activities <u>today</u> (e.g. work, study, housekeeping, family and leisure activities)

- I have no problems doing my daily activities. ☐
- I have slight problems doing my daily activities. ☐
- I have moderate problems doing my daily activities. ☐
- I have severe problems doing my daily activities. ☐
- I am unable to do my daily activities. ☐

E7. Your pain/discomfort <u>before</u> the symptoms of your genetic heart muscle disease occurred

- I had no pain or discomfort. ☐
- I had slight pain or discomfort. ☐
- I had moderate pain or discomfort. ☐
- I had severe pain or discomfort. ☐
- I had extreme pain or discomfort ☐

E8. Your pain or discomfort <u>today</u>

- I have no pain or discomfort. ☐
- I have slight pain or discomfort. ☐
- I have moderate pain or discomfort. ☐
- I have severe pain or discomfort. ☐
- I have extreme pain or discomfort. ☐

© EuroQol Research Foundation. EQ-5D™ is a trade mark of the EuroQol Research Foundation

E9. Your anxiety/depression before the symptoms of your genetic heart muscle disease occurred

- I was not anxious or depressed. ☐
- I was slightly anxious or depressed. ☐
- I was moderately anxious or depressed. ☐
- I was severely anxious or depressed. ☐
- I was extremely anxious or depressed. ☐

E10. Your anxiety or depression today

- I am not anxious or depressed. ☐
- I am slightly anxious or depressed. ☐
- I am moderately anxious or depressed. ☐
- I am severely anxious or depressed. ☐
- I am extremely anxious or depressed. ☐

E11. How would you rate your self-esteem before your symptoms of your genetic heart muscle disease occurred on a scale of 1 to 5, with 1 being low self- esteem and 5 being high self-esteem?

- 1 ☐
- 2 ☐
- 3 ☐
- 4 ☐
- 5 ☐

E12. How would you rate your self-esteem today on a scale of 1 to 5, with 1 being low self-esteem and 5 being high self-esteem?

- 1 ☐
- 2 ☐
- 3 ☐
- 4 ☐
- 5 ☐

© EuroQol Research Foundation. EQ-5D™ is a trade mark of the EuroQol Research Foundation

E13. We would like to know how good or bad your health was BEFORE the symptoms of your genetic heart disease occurred.

- This scale is numbered from 0 to 100.
- 100 means the best health you can imagine. 0 means the worst health you can imagine.
- Place an X on the scale to indicate how your health was BEFORE your first symptoms
- Now write down the number you indicated on the measuring scale in the corresponding box:

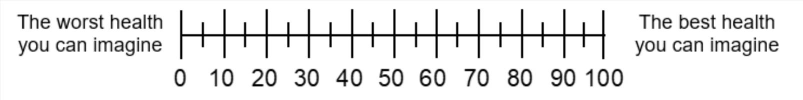

E14. We would like to know how good or bad your health is TODAY.

- This scale is numbered from 0 to 100.
- 100 means the best health you can imagine. 0 means the worst health you can imagine.
- Place an X on the scale to indicate how your health is today
- Now write down the number you indicated on the measuring scale in the corresponding box:

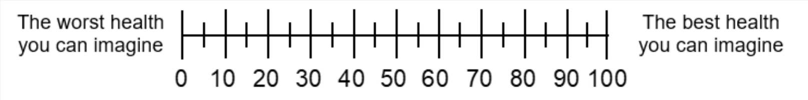

Section F: Social relationships

This part of the survey focuses on your social relationships and the influence of your illness on these relationships. We are interested in how your condition has affected your interactions with family, friends and other social contacts.

F1. Has your illness influenced your desire to have children?

- Yes ☐
- No ☐
- My desire to have children was already fulfilled before I received the diagnosis ☐
- I don't want to have children for reasons unrelated to my illness. ☐
- I do not know ☐

F2. If so, in which way? (Multiple answers possible)

- I now want more children than before ☐
- I now want fewer children than before ☐
- I would like to have (more) children, but I was advised not to get pregnant (anymore) because of my illness ☐
- I am considering IVF-PGT (Pre-implantation Genetic Testing - specialized technique that can detect a genetic abnormality in an embryo) as an option ☐
- I am considering other options such as adoption ☐
- I don't want any more children ☐
- I do not know ☐

Other ☐

Other

[ ]

F3. **Do you sometimes experience any form of stigmatization, i.e. expectations of prejudice, stereotyping and discrimination, because of your illness?**

- Very often ☐
- Often ☐
- Occasionally ☐
- Rarely ☐
- Never ☐
- I do not know ☐

F4. **Has your health condition caused problems with your:**

	Yes	Uncertain	No
Social life? (going out, meeting friends, going to the movies, etc.)	☐	☐	☐
Householding? (cleaning, cooking, chores, etc.)	☐	☐	☐
Home life? (relationship with housemates, etc.)	☐	☐	☐
Sex life?	☐	☐	☐
Interests and hobbies? (sports, crafts, DIY, etc.)	☐	☐	☐
Holidays? (summer or winter holidays, weekend getaways, etc.)	☐	☐	☐

Section G: Other conditions

This section of the survey examines possible other health conditions you might experience in addition to your genetic heart muscle disease. We are interested in obtaining a comprehensive picture of your overall health situation and any associated conditions that may impact your well-being.

G1. **Do you suffer from any of the following conditions that may be associated with your genetic heart muscle disease?**

	Yes	Uncertain	No
Heart failure	☐	☐	☐
Heart rhythm irregularities	☐	☐	☐
Cardiac arrest (status after resuscitation)	☐	☐	☐

G2. How often do you experience heart rhythm irregularities?

- Always ☐
- 1-2 times per week ☐
- 2-3 times per month ☐
- Once per month ☐
- Once every two months (6 times per year) ☐
- Once every three months (4 times per year) ☐
- 1-2 times per year ☐
- Never/less than once per year ☐
- I do not know ☐

G3. Do you suffer from any additional health problems not related to your genetic heart muscle disease?

- Yes ☐
- No ☐
- I do not know ☐

G4. If yes, please indicate those conditions in the list below. You can check more than one box. Also, if known, indicate the specific name of the additional condition it in the corresponding text box. If you do not yet know your diagnosis, you can indicate this at the bottom. (multiple answers possible)

Heart or blood vessel disease ☐

Comment

Respiratory tract disease ☐

Comment

Digestive tract disease ☐

Comment

Skin disease ☐

Comment

Disease of the locomotor system (bones, joints, muscles)

Comment

Mental health disorders

Comment

Hematological disorders (blood) or immune diseases

Comment

Endocrine, nutritional or metabolic disorders

Comment

Nervous system disease

Comment

Eye disease and its appendages

Comment

Disease of the ear or vestibular system

Comment

Disease of the urogenital system

Comment

Multi-system disease

Comment

No known diagnosis ☐

Comment

☐ Does not apply

Comment

Other ☐

Other

Section H: Use of care and accessibility of care

This part of the survey examines your experiences with the use of healthcare services and the accessibility of care. We are interested in your successes and challenges in obtaining care.

H1. Within the last 12 months, were there any instances where you needed care for your genetic heart diseases, but didn't receive it? This could be treatment, consultation, medical tests, rehabilitation, etc.

Yes ☐

No ☐

I do not know ☐

H2. If yes, for what reason(s) did you not receive the care you needed? (multiple answers possible)

Distance between home and place of care ☐

Transport problems (no public transport nearby, no personal vehicle, unable to drive own vehicle, no one to take you to your care) ☐

Lack of competent staff to give you the care you need ☐

Very long waiting times ☐

Difficulties in paying for care ☐

Fear of medical tests, hospital or other ☐

Lack of time because of work, childcare or other ☐

Lack of information ☐

Language problems ☐

Other ☐

Other

H3. What type of care provider(s) have you been in contact with due to your genetic heart muscle disease? (Multiple answers possible)

General practitioner ☐

Emergency Department Team ☐

Team of revalidation centre ☐

Physiotherapist ☐

Nurse at home ☐

Psychologist ☐

Social worker ☐

Specialist doctor ☐

Other ☐

Other

Section I: Treatment related to your genetic heart muscle disease

This section of the survey focuses on the treatments you are receiving for your health condition(s). We are interested in the different types of treatments you have undergone, their effectiveness, any side effects and your general satisfaction with the care received.

I1. Are you being or have you been treated, or are you following or have you followed specific advice from your doctor for your genetic heart muscle disease?

Yes ☐

No ☐

I do not know ☐

I2. What treatments are you receiving (or have you received) for your genetic heart muscle disease? And how satisfied are you with these?

	Very satisfied	Rather satisfied	Rather unsatisfied	Very unsatisfied	I don't get (or didn't get) this kind of treatment
A prescription drug	☐	☐	☐	☐	☐
An over-the-counter medicine	☐	☐	☐	☐	☐
Physiotherapy	☐	☐	☐	☐	☐
Medical device (e.g. ICD, pacemaker)	☐	☐	☐	☐	☐
Surgical intervention (e.g. Heart transplant, catheter ablation)	☐	☐	☐	☐	☐
Lifestyle adjustment (e.g. healthy eating, maintaining a healthy	☐	☐	☐	☐	☐
Stop competitive sports	☐	☐	☐	☐	☐

I3. Which medicines have you taken your genetic heart muscle disease within the past two years? (multiple answers possible)

- ☐ Beta blockers: Metoprolol (©Selokeen ZOK), Bisoprolol (©Emcor), Nebivolol (©Nebilet)
- ☐ Sotalol (©Sotacor)
- ☐ Amiodarone (©Cordarone)
- ☐ Flecainide (©Tambocor)
- ☐ Angiotensin-converting enzyme 1 inhibitors (ACE-I): Perindopril erbumine (©Coversyl)
- ☐ Angiotensin receptor blockers (ARBs): Losartan (©Cozaar), Valsartan (©Diovan)
- ☐ Sacubitril/ Valsartan (©Entresto)
- ☐ SGLT2 inhibitors: Dapagliflozin (©Forxiga)
- ☐ Diuretics / Water pills: Furosemide (©Lasix), Torasemide (©Sandoz)
- ☐ Mineralocorticoid receptor antagonists: Eplerenone (©Inspra), Spironolactone (©Aldactone)
- ☐ Statins: Atorvastatin (©Lipitor), Simvastatin (©Zocor), Rosuvastatin (©Crestor), Pravastatin (©Pravachol)
- ☐ I do not know
- ☐ Other ▼

Other

[ ]

I4. What surgical procedure(s) have you had for your genetic heart muscle disease? (Multiple answers possible)

- ☐ Implantable cardioverter defibrillator (ICD)
- ☐ Heart transplant

Left Ventricular Assist Device (LVAD) ☐

Catheter ablation ☐

Cardiac resynchronization therapy ☐

Cardiac denervation (LCSD) ☐

I do not know ☐

None ☐

Other ☐

Other

I5. How burdensome has the treatment for your genetic heart muscle disease been?

Extremely burdensome ☐

Quite burdensome ☐

Slightly burdensome ☐

Not burdensome ☐

Don't know ☐

I6. If the treatment was burdensome, for what reason(s)? (multiple answers possible)

Because of the side effects ☐

Because it forces me to constantly manage my illness/ treatment ☐

Because it demands a very strict life discipline (taking medication at fixed times, hygiene...) ☐

Because I have been on the this treatment(s) for a long time ☐

I do not know ☐

Other ☐

Other

I7. If you experience(d) any side effects when treating your genetic heart muscle disease, indicate to what extent each of these are/ were disturbing.

	Very disturbing	Rather disturbing	Slightly disturbing	Not at all disturbing	I have/didn't suffer from this side effect
Vomiting	☐	☐	☐	☐	☐
Nausea	☐	☐	☐	☐	☐
Constipation	☐	☐	☐	☐	☐
Diarrhoea	☐	☐	☐	☐	☐
Kidney insufficiency/problems	☐	☐	☐	☐	☐
Weight gain	☐	☐	☐	☐	☐
Thyroid problems	☐	☐	☐	☐	☐
Heart rhythm irregularities	☐	☐	☐	☐	☐
Fatigue or exhaustion	☐	☐	☐	☐	☐
Complication(s) of the procedure	☐	☐	☐	☐	☐
Unexpected shocks from an ICD	☐	☐	☐	☐	☐

I8. Did you experience any other side effects from the treatment(s)? If so, what were they and how disturbing were they to you?

I9. Do you think the treatment(s) you have received for your genetic heart muscle diseas has been effective?

- Yes ☐
- Yes, but only for a certain period of time ☐
- Yes, to a certain extent ☐
- No ☐
- I don't know ☐

I10. How would you describe your adherence?

(Adherence means taking your medication as prescribed by your doctor, at the right time and in the right dose)

- I always take my medication as prescribed by my doctor. ☐
- I usually take my medication as prescribed by my doctor. ☐
- I sometimes take medication as prescribed by my doctor. ☐
- I never take my medication as prescribed by my doctor. ☐

Section J: Information on your genetic heart muscle disease

This part of the survey examines the information you have sought or received about your health condition(s). We are interested in where you found this information, how useful and understandable it was, and whether it contributed to your understanding of your condition and the treatments available.

J1. To what extent do you receive useful information about your disease and its treatment from your healthcare providers at the time you need it to manage your health and well-being?

- I receive too much useful information ☐
- I always receive enough useful information ☐
- I often receive enough useful information ☐
- I sometimes receive enough useful information ☐
- I do not receive any useful information ☐
- Not relevant ☐
- I do not know ☐

J2. Where did you get the most useful support and information? (Mulitple answers possible)

- Doctor or specialist ☐
- Medical websites or apps ☐
- Patient organization ☐
- Facebook groups ☐
- Other patients ☐
- Other ☐

Other

J3. Would you like or would you have liked to be more involved in the choices about your treatment(s) for your genetic heart muscle disease?

- Yes ☐
- No ☐
- I do not know ☐

Section K: Financial impact

This section of the survey examines the financial impact your illness has had.

K1. Has your genetic heart muscle disease had a financial impact?

- Yes ☐
- No ☐
- I don't kow ☐

K2. If yes, what kind of impact? (Several answers possible)

- Loss or lack of income ☐
- Loss or lack of employment ☐
- Medical expenses ☐
- Other ☐

Other

Section L: Social Support network

This section of the survey examines your support network and the extent to which you have received logistical assistance with your daily activities, as well as the availability of a counselor to talk to regarding your health challenges.

L1. Do you need (or did you need) support due to your genetic heart muscle disease that you did not receive: (several answers possible)

- Talk to other patients with a genetic heart muscle disease ☐
- Support through a patient organization. ☐
- Talking to health professionals (e.g. psychologist) ☐
- Talking about things other than your health problems ☐
- More logistical assistance with daily activities than you currently receive ☐
- Get administrative or social assistance ☐
- Be accompanied by someone at a spiritual or religious level ☐
- Support when returning to work ☐
- No I did not need support ☐
- I do not know ☐

Other ☐

Other

[ ]

L2. Have you (had) difficulties finding someone you can trust to talk to about your genetic heart muscle disease?

Yes ☐

No ☐

I don't (didn't) need a trusted person to talk to about my genetic heart muscle disease ☐

I do not know ☐

L3. Are there one or more other important needs that you were unable to express in the questionnaire?

Yes ☐

No ☐

L4. If yes, which one(s)?

[ ]

Section M: Continuation of the study

If you have a heart muscle disease due to a hereditary predisposition in the PKP2 of the PLN gene, we would like to interview you. This interview will be about your needs, but also your preferences with important implications for your current treatm ent and future gene therapy. If you wish, you can participate in an individual interview (online or on-site with a researcher) to discuss this in more detail. This conversation will take place and is separate from the care you receive from your healthcare p rovider. The interview will last a maximum of one and a half hours.

M1. If you are interested in the interview, please provide us with your contact details (phone number and email address) so that we can contact you.

E-mail ☐

Confirm email ☐

☐

Mobile number ☐

M2. Are you involved in a patient association or discussion group (e.g. Facebook group) for patients with genetic heart muscle diseases?

Yes ☐

No ☐

I wish not to answer ☐

M3. What is the name of this association or discussion group?

☐

Dear participant,
Thank you for your interest in our questionnaire!

You may send the completed questionnaire by post using the enclosed envelope. This already contains a stamp.

If you have any further questions or would like to share more information, please do not hesitate to contact us at [X].

Supplementary material II: Additional tables

Table 1: *Sociodemographic characteristics of participants*

		n (%)
**Sex (n=112)**	*Women*	*63 (56,3)*
	Men	*49 (43,8)*
**Largest age group (years) (n=112)**	*18-25 years*	*6 (5.4)*
	25-30 years	*5 (4.5)*
	30-40 years	*11 (9.8)*
	40-50 years	*27 (24.1)*
	50-60 years	*26 (23.2)*
	60-70 years	*23 (20.5)*
	Older than 70 years	*14 (12.5)*
**Highest level of education (n=112)**	*No diploma*	*2 (1.8)*
	Secondary education	*32 (28.8)*
	HBO5	*6 (5.4)*
	Higher education of the short type(e.g. bachelor's degree)	*30 (27.0)*
	Higher education of the long type (e.g. master's)	*30 (27.0)*
	PhD	*7 (6.3)*
	Other	*4 (3.6)*
**Living situation (n=112)**	*Alone*	*20 (17,9)*
	Alone with children	*4 (3,6)*
	Together with partner, without children	*36 (32,1)*
	Together with partner and children	*42 (37,5)*
	With parents or family	*10 (8,9)*
**Occupation before diagnosis (n=112)**	*Employee (physical work)*	*26 (23,2)*
	Self-employed (physical work)	*4 (3,6)*
	Employee (office work)	*44 (39,3)*
	Unemployed	*1 (0,9)*
	Retired	*11 (9,8)*
	Student	*10 (8,9)*
	Incapacitated	*1 (0,9)*
	Other	*4 (3,6)*
**Occupation today (n=111)**	*Employee (physical work)*	*10 (9,0)*
	Self-employed (physical work)	*4 (3,6)*
	Employee (office work)	*39 (35,1)*
	Unemployed	*1 (0,9)*
	Retired	*29 (26,1)*

	Student	*7 (6,3)*
	Incapacitated	*12 (10,8)*
	Other	*2 (1,8)*
**Time since diagnosis (n=112)**	*More than 10 years*	*39 (34,8)*
	5 to 10 years	*25 (22,3)*
	2 to 5 years	*28 (25,0)*
	6 months to 2 years	*15 (13,4)*
	2 months to 6 months	*4 (3,6)*
	I do not know	*1 (0,9)*
**Experience of physical symptoms (n=112)**	*Yes*	*76 (67,9)*
	No	*36 (32,1)*
**Gene(s) responsible* (n=112)**	*PKP2*	*9 (8,0)*
	DSG2	*2 (1,8)*
	DSP	*20 (17,9)*
	DES	*4 (3,6)*
	PLN	*6 (5,4)*
	FLNC	*29 (25,9)*
	LMNA	*14 (12,5)*
	I do not know	*29 (25,9)*

** Multiple answer question.*

Table 2: *Self-reported level of problems on each EQ-5D-5L dimension of symptomatic carriers (S) (before onset and present, n=76) and asymptomatic carriers (A) (present, n=36).*

Level of problems	Carrier state (A/S)	Mobility (n, %)				Self-care (n,%)				Daily activities (n,%)				Pain/ discomfort (n, %)				Anxiety/ depression (n, %)			
		**BEFORE**		**PRESENT**		**BEFORE**		**PRESENT**		**BEFORE**		**PRESENT**		**BEFORE**		**PRESENT**		**BEFORE**		**PRESENT**	
**No problems**	S	*66*	*86,8*	*54*	*71,1*	*75*	*98,7*	*71*	*93,4*	*64*	*84,4*	*37*	*48,7*	*54*	*71,1*	*32*	*42,1*	*63*	*82,9*	*39*	*51,3*
	A			*32*	*88,9*			*35*	*97,2*			*33*	*91,7*			*30*	*83,3*			*25*	*69,4*
**Slight problems**	S	*5*	*6,6*	*10*	*13,2*	*0*	*0,0*	*3*	*3,9*	*10*	*12,9*	*20*	*26,3*	*15*	*19,7*	*27*	*35,5*	*8*	*10,5*	*21*	*27,6*
	A			*2*	*5,6*			*1*	*2,8*			*2*	*5,6*			*4*	*11,1*			*7*	*19,4*
**Moderate problems**	S	*3*	*3,9*	*6*	*7,9*	*1*	*1, 3*	*1*	*1,3*	*2*	*2,60*	*12*	*15,8*	*1*	*1,3*	*9*	*11,8*	*4*	*5,3*	*9*	*11,8*
	A			*2*	*5,6*			*0*	*0,0*			*1*	*2,8*			*2*	*5,6*			*3*	*8,3*
**Severe problems**	S	*0*	*0,0*	*4*	*5,3*	*0*	*0,0*	*1*	*1,3*	*0*	*0,0*	*3*	*3,9*	*5*	*6,6*	*6*	*7,9*	*1*	*1,3*	*5*	*6,6*
	A			*0*	*0,0*			*0*	*0,0*			*0*	*0,0*			*0*	*0,0*			*1*	*2,8*
**Extreme problems**	S	*2*	*2,6*	*2*	*2,6*	*0*	*0,0*	*0*	*0,0*	*0*	*0,0*	*4*	*5,3*	*1*	*1,3*	*2*	*2,6*	*0*	*0,0*	*2*	*2,6*
	A			*0*	*0,0*			*0*	*0,0*			*0*	*0,0*			*0*	*0,0*			*0*	*0,0*

Table 3: *Change in self-reported level of problems (% before onset - % present)) on each EQ-5D-5L dimension of symptomatic carriers (n=76).*

Level of problems	Mobility	Self-care	Daily activities	Pain/ discomfort	Anxiety/ depression
**No problems**	*15,7*	*5,3*	*35,07*	*29;0*	*31,6*
**Slight problems**	*-6,6*	*-3,9*	*-13,3*	*-15,8*	*-17,1*
**Moderate problems**	*-4,0*	*0*	*-13,2*	*-10,5*	*-6,5*
**Severe problems**	*-5,30*	*-1,3*	*-3,9*	*-1,3*	*-5,3*
**Extreme problems**	*0*	*0*	*-5,3*	*-1,3*	*-2,6*
**Total problem %**	*-15,90*	*-5,2*	*-35,7*	*-28,9*	*-31,5*

Table 4: *Mean EQ-5D-5L health index scores of symptomatic carriers (before onset and present, n=76) and asymptomatic carriers (present, n=36).*

Group	Mean	SD	95%CI	P-value
**Symptomatic carriers (before onset)**	*0,9059*	*0,16736*	*0,8676; 0,9441*	
**Symptomatic carriers (present)**	*0,7489*	*0,33424*	*0,6725; 0,8252*	
**Asymptomatic carriers (present)**	*0,9314*	*0,10844*	*0,8947; 0,9681*	
**Beta ***	*-0,1570*	*0,34413*	*-0,2357; -0,0784*	*<0,001*

**Beta= [EQ-5D-5L Symptomatic carriers (present)] – [EQ-5D-5L Symptomatic carriers (before onset)]*

Table 5: *Self-reported frequency and level of disturbance of physical symptoms (among respondents reporting physical symptoms, n= 75/ 76).*

Symptoms	Frequency (n,%)		Reported burden (n, %)							
			Not at all disturbing		Slightly disturbing		Rather disturbing		Very disturbing	
**Reduced fitness and endurance**	*66*	*88,0*	*7*	*10,4%*	*14*	*20,9%*	*25*	*37,3%*	*21*	*31,3%*
**Fatigue or exhaustions**	*67*	*88,2*	*6*	*9,0%*	*12*	*17,9%*	*27*	*40,3%*	*22*	*32,8%*
**Lack of energy**	*61*	*80,3*	*2*	*3,3%*	*17*	*27,9%*	*22*	*36,1%*	*20*	*32,8%*
**Palpitations**	*61*	*80,3*	*6*	*9,7%*	*16*	*26,2%*	*24*	*39,3%*	*15*	*24,6%*
**Light-headed feeling**	*55*	*72,4*	*8*	*14,5%*	*22*	*40,0%*	*19*	*34,5%*	*6*	*10,9%*
**Shortness of breath**	*51*	*67,1*	*6*	*11,8%*	*11*	*21,6%*	*22*	*43,1%*	*12*	*23,5%*
**Chest pain**	*34*	*44,7*	*8*	*23,5%*	*13*	*38,2%*	*6*	*17,6%*	*7*	*20,6%*
**Pass out**	*29*	*38,2*	*8*	*27,6%*	*7*	*24,1%*	*9*	*31,0%*	*5*	*17,2%*
**Oedema**	*30*	*39,5*	*5*	*16,7%*	*14*	*46,7%*	*5*	*16,7%*	*6*	*20,0%*

Table 6: *Self-reported frequency and level of disturbance of psychological symptoms (among respondents reporting psychological symptoms, n=66)*
).

Symptoms	Frequency (n,%)		Reported burden (n, %)							
			Not at all disturbing		Slightly disturbing		Rather disturbing		Very disturbing	
**Fear/ anxiety**	*58*	*87,9%*	*8*	*13,8%*	*18*	*31,0%*	*18*	*31,0%*	*14*	*24,1%*
**Stress**	*54*	*81,8%*	*7*	*13,0%*	*17*	*31,5%*	*16*	*29,6%*	*14*	*25,9%*
**Feelings of restlessness and/ or nervousness**	*53*	*80,3%*	*14*	*26,40%*	*15*	*28,3%*	*15*	*28,3%*	*9*	*17,0%*
**Feeling down or depressed**	*50*	*75,8%*	*7*	*14,00%*	*17*	*34,0%*	*17*	*34,0%*	*9*	*18,0%*
**Concentration problems**	*44*	*66,7%*	*7*	*15,90%*	*12*	*27,3%*	*18*	*40,9%*	*7*	*15,9%*
**Mood changes**	*41*	*62,1%*	*8*	*19,50%*	*19*	*46,3%*	*6*	*14,6%*	*8*	*19,5%*
**Anger/ aggressiveness**	*23*	*34,8%*	*8*	*34,80%*	*8*	*34,8%*	*5*	*21,7%*	*2*	*8,7%*

Table 7: *Impact of ACM on reproductive decision-making of participants and underlying reasons.*

		n (valid %)		
		Total	*S (n=75)*	*A (n=36)*
**Influence on desire to have children (n=111)**	*Yes*	*13 (11,7)*	*8 (10,7)*	*5 (13,9)*
	No	*43 (38,7)*	*25 (33,3)*	*18 (50,0)*
	Desire for children fulfilled before diagnosis	*50 (45,0)*	*39 (52,0)*	*11 (30,6)*
	No desire for children unrelated to condition	*2 (1,8)*	*2 (2,7)*	*0 (0,0)*
	I do not know	*3 (2,7)*	*1 (1,3)*	*2 (5,6)*
**Reasons of influence (n=14)**	*Want more children, but advised against pregnancy due to condition*	*3 (21,4)*	-	-
	Consideration of IVF-PGT	*7 (50,0)*	-	-
	Consideration of other options (e.g. adoption)	*1 (7,1)*	-	-
	No desire for more children due to condition	*4 (28,6)*	-	-
	Uncertain	*2 (14,3)*	-	-
	Other	*2 (14,3)*	-	-

S: Symptomatic carriers, A: Asymptomatic carriers, IVF-PGT: In Vitro Fertilization Preimplantation Genetic Testing

Table 8: *Self-reported frequency and level of satisfaction of treatment strategies (n=94).*

Symptoms	Frequency (n,%)		Reported satisfaction (n, %)							
			Very unsatisfied		Rather unsatisfied		Rather satisfied		Very satisfied	
**An over-the-counter medicine**	*17*	*18,20%*	*0*	*0,0%*	*0*	*0,0%*	*7*	*41,2%*	*10*	*58,8%*
**Physiotherapy**	*19*	*20,20%*	*0*	*0,0%*	*0*	*0,0%*	*5*	*26,3%*	*14*	*73,7%*
**Stop competitive sports**	*42*	*45,20%*	*6*	*14,3%*	*15*	*35,7%*	*15*	*35,7%*	*6*	*14,3%*
**Surgical intervention**	*50*	*53,20%*	*2*	*4,0%*	*2*	*4,0%*	*9*	*18,0%*	*37*	*74,0%*
**Medical device**	*66*	*70,20%*	*0*	*0,0%*	*1*	*1,5%*	*12*	*18,2%*	*53*	*80,3%*
**Lifestyle adjustment**	*67*	*71,30%*	*2*	*3,0%*	*5*	*7,5%*	*28*	*41,8%*	*32*	*47,8%*
**A prescription drug**	*82*	*87,20%*	*0*	*0,0%*	*2*	*2,4%*	*30*	*36,6%*	*50*	*61,0%*

Table 9: *Frequency of surgical interventions over disease course and drugs usage in past two years.*

		n (valid %)
**Surgical interventions* (n=74)**	*Implantable cardioverter Defibrillator*	*47 (63,5)*
	Heart transplant	*17 (23,0)*
	Left Ventricular Assist Device	*5 (6,8)*

	Catheter Ablation	*29 (39,2)*
	Cardiac resynchronization therapy	*4 (5,4)*
	Cardiac denervation	*1 (1,4)*
	Other	*4 (5,4)*
**Drugs* (n=82)**	*Beta-blockers*	*59 (72,0)*
	Sotalol	*7 (8,5)*
	Amiodarone	*16 (19,5)*
	Flecainide	*8 (9,8)*
	Angiotensin-converting enzyme 1 inhibitors	*14 (17,1)*
	Angiotensin receptor blockers)	*5 (6,1)*
	Sacubitril/ valsartan	*18 (22,0)*
	SGLT2 inhibitors	*14 (17,1)*
	Diuretic/ water pills	*11 (13,4)*
	Mineralocorticoid receptor antagonist	*20 (24,4)*
	Statines	*30 (36,6)*
	Uncertain	*6 (7,3)*
	Other	*28 (34,1)*

*SGLT: sodium-dependent glucose cotransporters, * multiple answer question.*

Table 10: *Self-reported treatment effectiveness, burden and adherence.*

		n (valid %)
**Perceived effectiveness of treatment (n=93)**	*Yes*	*46 (49,5)*
	Yes, but only for a certain period of time	*6 (6,5)*
	No	*2 (2,2)*
	Uncertain	*15 (16,1)*
	Yes, to a certain extent	*24 (25,8)*
**Level of burdensomeness treatment (n=94)**	*Extremely burdensome*	*9 (9,6)*
	Quite burdensome	*12 (12,8)*
	Slightly burdensome	*26 (27,7)*
	Not burdensome	*40 (42,6)*
	I do not know	*7 (7,4)*
**Reason of burdensomeness (n=54)**	*Because of side effects*	*25 (46,3)*
	Because it forces me to constantly manage my illness/ treatment	*21 (38,9)*
	Because it demands a very strict life discipline (asking medication at fixed times, hygiene,…)	*19 (35,2)*
	Because I have been on this treatment(s) for a long time	*13 (24,1)*

	I do not know	*1 (1,9)*
	Other	*13 (11,6)*
**Self-reported treatment adherence (n=93)**	*I always take my medication as prescribed by my doctor.*	*80 (85,1)*
	I usually take my medication as prescribed by my doctor.	*11 (11,8)*
	I sometimes take my medication as prescribed by my doctor.	*1 (1,1)*
	I never take my medication as prescribed by my doctor.	*1 (1,1)*

Table 11: *Self-reported frequency and level of burden of side-effects (n=94).*

Symptoms	Frequency (n,%)		Reported burden (n, %)							
			Not at all disturbing		Slightly disturbing		Rather disturbing		Very disturbing	
**Vomiting**	*11*	*11,70%*	*2*	*25,0%*	*1*	*8,3%*	*4*	*33,3%*	*4*	*33,3%*
**Nausea**	*20*	*21,30%*	*4*	*20,0%*	*1*	*5,0%*	*9*	*45,0%*	*6*	*30,0%*
**constipation**	*16*	*17,00%*	*3*	*23,5%*	*4*	*23,5%*	*6*	*35,3%*	*3*	*17,6%*
**Diarrhoea**	*19*	*20,20%*	*4*	*25,0%*	*3*	*15,0%*	*7*	*35,0%*	*5*	*25,0%*
**Kidney insufficiency**	*14*	*14,90%*	*4*	*33,3%*	*3*	*20,0%*	*6*	*40,0%*	*1*	*6,7%*
**Weight gain**	*27*	*28,70%*	*1*	*3,7%*	*5*	*18,5%*	*10*	*37,0%*	*11*	*40,7%*
**Thyroid problems**	*13*	*13,80%*	*2*	*21,4%*	*4*	*28,6%*	*4*	*28,6%*	*3*	*21,4%*
**Heart rhythm irregularities**	*48*	*51,10%*	*6*	*12,5%*	*10*	*20,8%*	*17*	*35,4%*	*15*	*31,3%*
**Fatigue or exhaustion**	*61*	*64,90%*	*4*	*8,1%*	*11*	*17,7%*	*23*	*37,1%*	*23*	*37,1%*
**Complication(s) f the procedure**	*24*	*25,50%*	*2*	*8,3%*	*7*	*29,2%*	*7*	*29,2%*	*8*	*33,3%*
**Unexpected shocks from an ICD**	*21*	*22,30%*	*2*	*9,5%*	*4*	*19,0%*	*4*	*19,0%*	*11*	*52,4%*

Table 12: *Type of healthcare provides participants have been in contact with for their ACM.*

		n (valid %)
**Types of care providers (n=112)**	*General practitioner*	*84 (75,0)*
	Emergency department	*37 (33,0)*
	Team of revalidation centre	*15 (13,4)*
	Physiotherapist	*20 (17,9)*
	Nurse at home	*5 (4,5)*
	Psychologist	*19 (17,0)*
	Social worker	*7 (6,3)*
	Specialist doctor	*102 (91,1)*
	Other	*2 (1,8)*

Table 13: *Receival of useful information at the moment carriers need it and the source of most useful information.*

		n (valid %)		
		Total	*S (n= 75/ 76)*	*A (n=36)*
**Receival of useful information (n=111)**	*I do not receive any useful information*	*12 (10,8)*	*7 (9,3)*	*5 (13,9)*
	I sometimes receive enough useful information	*18 (16,2)*	*13 (17,3)*	*5 (13,9)*
	I often receive enough useful information	*26 (23,4)*	*19 (25,3)*	*7 (19,4)*
	I always receive enough useful information	*45 (40,5)*	*34 (45,3)*	*11 (30,6)*
	I receive too much useful information	*4 (3,6)*	*0 (0,0)*	*4 (11,1)*
	Not relevant	*5 (4,5)*	*1 (1,3)*	*4 (11,1)*
	Uncertain	*1 (0,9)*	*1 (1,3)*	*0 (0,0)*
**Source of most useful information (n=112)**	*Doctor or specialist*	*101 (90,2)*	*70 (92,1)*	*5 (86,1)*
	Medical websites or apps	*20 (17,9)*	*16 (21,1)*	*4 (11,1)*
	Patient organisation	*3 (2,7)*	*3 (3,9)*	*0 (0,0)*
	Facebook groups	*1 (0,9)*	*1 (1,3)*	*0 (0,0)*
	Other patients	*10 (8,9)*	*7 (9,2)*	*3 (8,3)*
	Other	*9 (8,0)*	*5 (6,6)*	*4 (11,1)*

S: Symptomatic carriers, A: Asymptomatic carriers.

Table 14: *Pathway and timing to diagnosis and treatment.*

		n (valid %)
**Way of discovery heart condition (n=112)**	*I visited my doctor after experiencing symptoms related to the disease.*	*26 (23,2)*
	Through genetic screening after a family member was diagnosed with the disease.	*52 (46,4)*
	An abnormality in my heart was accidentally discovered during a medical check-up or a regular doctor's appointment.	*3 (2,7)*
	An abnormality in my heart was discovered after an emergency admission to a hospital.	*19 (17,0)*
	Other	*12 (10,7)*
**Time first symptoms – decision (n=24)**	*Less than 1 week*	*7 (29,2)*
	1 week to 1 month	*6 (25,0)*
	1 month to 3 months	*7 (29,2)*
	More than 3 months	*4 (16,7)*

**Time decision – appointment (n=24)**	*Less than 1 week*	*12 (50,0)*
	1 week to 1 month	*8 (33,3)*
	1 month to 3 months	*2 (8,3)*
	I do not know	*2 (8,3)*
**Time appointment – diagnosis (n=107)**	*Less than 2 weeks*	*19 (17,8)*
	2 weeks to 2 months	*25 (23,4)*
	2 months to 1 year	*22 (20,6)*
	I do not know	*13 (12,1)*
	More than 1 year	*28 (26,2)*
**Time diagnosis – treatment (n=79)**	*Less than 2 weeks*	*34 (43,0)*
	2 months to 1 year	*12 (15,2)*
	I do not know	*8 (10,1)*
	2 weeks to 2 months	*22 (27,8)*
	More than 1 year	*3 (3,8)*

Table 15: *Reported need for additional support that was not received (n=112).*

	**n (valid %)**		
	Total	**S (n=75)**	**A (n=36)**
Talk to other patients with a genetic heart muscle disease	*18 (16,2)*	*15 (20,0)*	*3 (8,3)*
Support through a patient organization	*6 (5,4)*	*6 (8,0)*	*0 (0,0)*
Talking to health professionals (e.g. psychologist)	*21 (18,9)*	*17 (22,7)*	*4 (11,1)*
Talking about other than your health problems	*5 (4,5)*	*5 (6,7)*	*0 (0,0)*
More logistical assistance with daily activities than you currently receive	*2 (1,8)*	*2 (2,7)*	*0 (0,0)*
Get administrative or social assistance	*4 (3,6)*	*4 (5,3)*	*0 (0,0)*
Be accompanied by someone at a spiritual or religious level	*1 (0,9)*	*1 (1,3)*	*0 (0,0)*
Support when returning to work	*5 (4,5)*	*5 (6,7)*	*0 (0,0)*
No need of additional support	*57 (51,4)*	*32 (42,7)*	*25 (69,4)*
Uncertain	*13 (11,7)*	*10 (13,3)*	*3 (8,3)*
Other	*7 (6,3)*	*7 (9,3)*	*0 (0,0)*

S: Symptomatic carriers, A: Asymptomatic carriers

Table 16: *Areas of NHP domains were participants experienced problems with (n=112).*

**n (valid %)**	**YES**			**UNCERTAIN**			**NO**		
	Total	***S***	***A***	***Total***	***S***	***A***	***Total***	***S***	***A***
Social life? (going out, meeting friends, going to the movies, etc.)	*30 (26,8)*	*28 (36,8)*	*2 (5,6)*	*3 (2,7)*	*1 (1,3)*	*2 (5,6)*	*79 (70,5)*	*47 (61,8)*	*32 (88,9)*

Householding? (cleaning, cooking, chores, etc.)	*34 (30,4)*	*32 (42,1)*	*2 (5,6)*	*2 (1,8)*	*1 (1,3)*	*1 (2,8)*	*76 (67,9)*	*43 (56,6)*	*33 (91,7)*
Home life? (relationships with housemates, etc.)	*21 (18,8)*	*20 (26,3)*	*1 (2,8)*	*3 (2,7)*	*2 (2,6)*	*1 (2,8)*	*88 (78,6)*	*54 (71,1)*	*34 (94,4)*
Sex life?	*24 (21,4)*	*23 (30,3)*	*1 (2,8)*	*9 (8,0)*	*7 (9,2)*	*2 (5,6)*	*79 (70,5)*	*46 (60,5)*	*33 (91,7)*
Interest and hobbies?	*53 (47,3)*	*45 (59,2)*	*8 (22,2)*	*3 (2,7)*	*1 (1,3)*	*2 (5,6)*	*56 (50,0)*	*30 (39,5)*	*26 (72,2)*
Holidays? (summer or winter holidays, weekend getaways, etc.)	*35 (31,3)*	*32 (42,1)*	*3 (8,3)*	*6 (5,4)*	*5 (6,6)*	*1 (2,8)*	*71 (63,4)*	*39 (51,3)*	*32 (88,9)*

S: Symptomatic carriers (n=76), A: Asymptomatic carriers (n=36).

Table 17: *Impact on career choice and work intensity.*

		n (valid %)		
		Total	*S (n=75/ 76)*	*A (n=36)*
**Influence ACM on career choice (n=112)**	*Yes*	*20 (17,9)*	*19 (25,0)*	*1 (2,8)*
	No	*87 (77,7)*	*54 (71,1)*	*33 (91,7)*
	I do not know	*5 (4,5)*	*3 (3,9)*	*2 (5,6)*
**Reasons of influence (n=25)**	*I have accepted a lower-paying position*	*1 (4,0)*	-	-
	I ended up in a different sector than I originally planned	*3 (12,0)*	-	-
	I chose a profession with flexible hours to better manage my health	*2 (8,0)*	-	-
	I chose a profession that is less physically demanding	*4 (16,0)*	-	-
	I do not know	*3 (12,0)*	-	-
	Other	*9 (36,0)*	-	-
**Influence ACM on work intensity (n=111)**	*Yes*	*40 (36,0)*	*36 (48,0)*	*4 (11,1)*
	No	*67 (60,4)*	*38 (50,7)*	*29 (80,6)*
	I do not know	*4 (3,6)*	*1 (1,3)*	*3 (8,3)*
**Reasons of influence (n=43)**	*Reducing of working hours*	*18 (41,9)*	-	-
	Frequently taking unpaid	*3 (7,0)*	-	-
	Frequently taking sick leave	*4 (9,3)*	-	-
	Loss of employment	*3 (7,0)*	-	-
	Taking early retirement	*5 (11,6)*	-	-
	I do not know	*5 (11,6)*	-	-
	Other	*9 (22,0)*	-	-

S: Symptomatic carriers, A: Asymptomatic carriers

Table 18: *Reported financial impact and reasons of impact.*

		n (valid %)		
		Total	*S (n=76)*	*A (n=35)*
**Financial consequence of condition (n=111)**	*Yes*	*39 (35,1)*	*36 (47,4)*	*3 (8,6)*
	No	*67 (60,4)*	*37 (48,7)*	*30 (85,7)*
	I do not know	*5 (4,5)*	*3 (3,9)*	*2 (5,7)*
**Reasons of consequence (n=44)**	*Loss or lack of income*	*24 (54,5)*	-	-
	Loss or lack of employment	*15 (34,1)*	-	-
	Medical expenses	*24 (54,5)*	-	-
	Other	*11 (25,0)*	-	-

S: Symptomatic carriers, A: Asymptomatic carriers

Table 19: *Reported experience of stigmatization*

	n (valid %)		
	Total	*S (n=76)*	*A (n=35)*
Rarely	*22 (19,8)*	*18 (23,7)*	*4 (11,4)*
Never	*56 (50,5)*	*33 (43,4)*	*23 (65,7)*
I do not know	*10 (9,0)*	*7 (9,2)*	*3 (8,6)*
Often	*7 (6,3)*	*5 (6,6)*	*2 (5,7)*
Very often	*2 (1,8)*	*2 (2,6)*	*0 (0,0)*
Occasionally	*14 (12,6)*	*11 (14,5)*	*3 (8,6)*

S: Symptomatic carriers, A: Asymptomatic carriers

Table 20: *Years of education lost due to ACM*

	n (valid %)		
	Total	*S (n=71)*	*A (n=34)*
Less than 6 months	*7 (6,7)*	*3 (4,2)*	*4 (11,8)*
6 months to 1 year	*3 (2,9)*	*3 (4,2)*	*0 (0,0)*
More than 2 years	*3 (2,9)*	*3 (4,2)*	*0 (0,0)*
I have not missed any education	*92 (87,6)*	*62 (87,3)*	*30 (88,2)*

S: Symptomatic carriers, A: Asymptomatic carriers
